# Modeling Within- and Between-Person Differences in the Use of the Middle Category in Likert Scales

**DOI:** 10.1177/01466216251322285

**Published:** 2025-03-02

**Authors:** Jesper Tijmstra, Maria Bolsinova

**Affiliations:** 1 7899 Tilburg University, The Netherlands

**Keywords:** item response theory, ordinal scales, Likert data, response styles, mixture models

## Abstract

When using Likert scales, the inclusion of a middle-category response option poses a challenge for the valid measurement of the psychological attribute of interest. While this middle category is often included to provide respondents with a neutral response option, respondents may in practice also select this category when they do not want to or cannot give an informative response. If one analyzes the response data without considering these two possible uses of the middle response category, measurement may be confounded. In this paper, we propose a response-mixture IRTree model for the analysis of Likert-scale data. This model acknowledges that the middle response category can either be selected as a non-response option (and hence be uninformative for the attribute of interest) or to communicate a neutral position (and hence be informative), and that this choice depends on both person- and item-characteristics. For each observed middle-category response, the probability that it was intended to be informative is modeled, and both the attribute of substantive interest and a non-response tendency are estimated. The performance of the model is evaluated in a simulation study, and the procedure is applied to empirical data from personality psychology.

## Introduction

Since almost all research in the social sciences in some way concerns person properties that are not and cannot directly be observed, answering the question of how to obtain valid measurements of such unobservable attributes plays (or should play) a crucial role in these scientific endeavors. One of the most common ways to obtain information about these latent attributes or traits is through the use of response data, obtained by having respondents provide answers to sets of questions that are taken to evoke responses that are indicative of the trait of interest.

Perhaps the most common and iconic format in which response data is obtained in the social sciences is the Likert scale (Cronbach, 1950; Likert, 1932). On Likert scales, respondents are presented with a statement or question, and are asked to communicate their position with respect to that statement using a limited set of prespecified response categories, which are usually accompanied by some kind of textual description conveying the intended meaning of those categories. While the responses are often coded numerically, the qualitative nature of the categories prevents an automatic interpretation of these item scores as being quantitative, as differences between adjacent response categories need not be of the same ‘quantity’ (however defined) for all categories. Hence, interval level measurement is not guaranteed for the item scores, and it is recommended to make use of analysis tools such as item response theory (IRT; see e.g., Embretson & Reise, 2013; Hambleton & Swaminathan, 1985; Lord & Novick, 1968) that do not rely on this assumption.

Despite their widespread application, the use of Likert scales is not without issues. One important issue concerns the qualitative nature of the response categories, which at least in principle allows respondents to differ in their interpretation as well as use of those categories (Kulas & Stachowski, 2009). While response options are often provided with a worded label (e.g., “strongly agree”), respondents can and often will differ in their exact interpretation of those labels. Additionally, persons who agree on the meaning of the labels may still decide to use those categories differently, giving rise to different response styles (e.g., see Baumgartner & Steenkamp, 2001). Such differences between persons in their interpretation and use of the categories complicates the analysis of their responses, as the selection of a particular category may provide different information depending on those differences. Unfortunately, these differences in interpretation and use are not directly observed, complicating efforts to take these differences into account in the measurement model for the attribute(s) of interest.

One of the most troublesome categories on Likert-scale items is the often-present neutral middle response category. Its inclusion or exclusion in Likert-scale items has been and continues to be debated in the literature (Kalton et al., 1980; Presser & Schuman, 1980; Raaijmakers et al., 2000; Sturgis et al., 2014). An important reason to include this category is that it captures the often relevant and valid possibility of holding a neutral stance towards the presented statement or question. Problematically, however, persons may differ in important ways in how they interpret and use this category. Some may use it to truly communicate a neutral position, but others may choose it due to not having taken a deliberated position (e.g., when the evaluated statement is too complex or difficult), or being unwilling to communicate their actual position (e.g., due to the sensitive nature of the question). Problematically, these different response processes result in the same observed outcome: the selection of the middle category. Ignoring this issue may confound measurement, and hence it is important to consider statistical tools for dealing with the differential use of the middle category.

In the context of response style modeling, a variety of statistical models have been proposed that allow for between-person differences in the usage of the middle response category. Generally, these response style methods either make use of latent classes (e.g., see Hernández et al., 2004; Maij-de Meij et al., 2008; Moors, 2008; Rost et al., 1997) or of an additional continuous latent variable (e.g., see Bolt et al., 2014; Falk & Cai, 2016; Tutz & Berger, 2016) to capture relevant differences in the tendency of persons to make use of the middle response category. In these models this latent variable is taken to either capture a specific midpoint response style (if the latent variable specifically concerns differences in the use of the middle category), or an extreme response style (if the latent variable influences the frequency with which more or less extreme categories are selected). While these models differ in their parametric form, they generally share the assumption that the middle category belongs to the same scale as the other categories and hence is still informative of the attribute of interest. That is, the item scores are still taken to be of ordinal measurement level.

**Figure 1.**
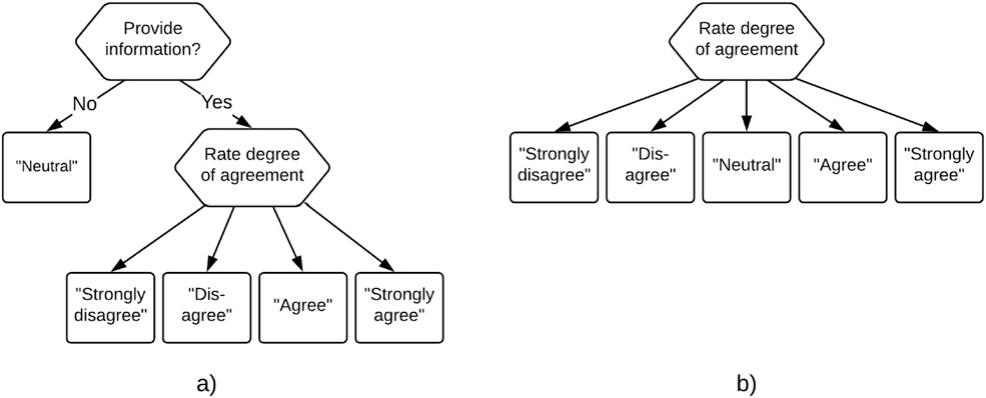
The two decision trees for a five-category Likert-scale item considered by Tijmstra et al. (2018), with (a) matching the non-informative usage of the middle response category and (b) matching the informative usage of the middle response category.

If one considers it plausible that some respondents use the middle response category as a non-response option, the assumption of ordinality of the item scores can no longer be maintained, as a middle-category response need not be informative of their location on the attribute of interest. One option to address this issue is by considering IRTree models (Böckenholt, 2012; Boeck & Partchev, 2012) that capture the non-response usage of the middle response category, such as the model proposed by Jeon and De Boeck (2016), which is displayed in Figure 1(a). Here, a two-step process is modeled using a decision tree, where in the first node respondents decide whether they will provide an informative response. If they do not want to give an informative response, they select the middle category and the process is terminated. If they do want to provide an informative response the process continues to the second node, where they select one of the *remaining* categories to communicate their degree of agreement.

A limitation of these IRTree models is that they exclude the possibility that some respondents do make use of the middle response category to give an informative response. Tijmstra et al. (2018) proposed to resolve this by considering a mixture model in which some respondents use the middle response category to give an informative response, while others use it as a non-response option. The responses of “normal” respondents can then be modeled using standard polytomous IRT models, while an IRTree model such as the one proposed by Jeon and De Boeck (2016) can be used to model the responses of respondents who use the middle response category as a non-response option. Thus, two latent classes of persons are considered, with the responses of persons in each class being modeled by structurally different models that match the differences in the response processes of these persons. The two corresponding decision trees are displayed in Figure 1.

While the mixture model of Tijmstra et al. (2018) does allow for differences between persons in how they approach the middle response category, an important limitation is that it assumes that persons will either always use that category in an informative way or always in an uninformative way. However, it is plausible that respondents frequently consider both the non-informative and informative use of the middle response category. On some items, they may feel that the middle response category best captures their position with respect to the statement in the question. On other items they may use the category as a non-response option, perhaps because they do not understand the question, do not have sufficient background knowledge to be able to answer it, or do not want to let their position on a sensitive topic be known. This is problematic for person-mixture approaches, as respondents are taken to either belong to the class of persons that exclusively uses the category in an informative way, or to the group that always uses it as a non-response option. As such they cannot account for within-person variation in how the middle category is used and measurement may still be confounded, as some informative responses are taken to be uninformative and vice versa.

**Figure 2.**
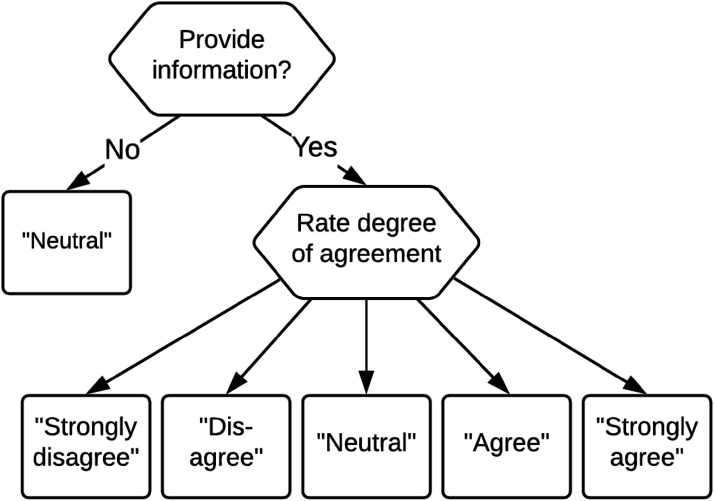
A decision tree for a five-category Likert-scale item with multiple terminal nodes for the middle response category.

In this paper, we propose a response-mixture IRTree approach for dealing with the two ways in which the middle response category can be used by respondents. The proposed IRTree model is based on the decision tree presented in Figure 2 and can be seen as a member of the hierarchical multinomial processing trees framework (Klauer, 2010), where some outcomes can be reached through different pathways in the tree. Such multinomial processing trees have been considered for the study of acquiescence (Plieninger & Heck, 2018) and extreme response styles (Thissen-Roe & Thissen, 2013), but to our knowledge has not been considered for the differential usage of the middle response category. Our decision tree merges the two trees of Tijmstra et al. (2018), and consequently differs from their person-mixture approach in three important ways. First, the response-mixture model acknowledges that all persons can use the middle response category both as a non-response option and as an informative response option. While persons will differ in their non-response tendency (i.e., the latent trait active in the first node), the non-response option is taken to in principle be available to all respondents. Second, as can be observed in Figure 2 there no longer is a one-to-one mapping of the observed outcome of the decision process to the path that was followed for all response options, as a middle-category response is obtained at two terminal nodes in the tree. Hence, for middle-category responses the path that is followed is unobserved and needs to be modeled, which requires response-mixture modeling for these responses.^1^ Third, for each observed middle-category response the probability that it was an informative response can be modeled, which will depend both on characteristics of the person (e.g., a non-response tendency) and the item (e.g., the amount of background information needed to answer the item, or the controversialness of the statement). This makes it possible to estimate the number of non-informative responses that a person has provided on a test, and likewise estimate the proportion of responses to an item that were non-informative.

The remainder of the paper is organized as follows. In Section 2, we introduce the response-mixture IRTree model. An estimation procedure using Markov chain Monte Carlo methods is proposed and discussed. Section 3 evaluates the performance of the estimation procedure in terms of parameter recovery, and compares the model’s bias and precision of the estimates of the latent trait to that of a standard polytomous IRT model. Section 4 illustrates the application of the procedure to empirical data from personality psychology. The paper concludes with a discussion.

## The Response-Mixture IRTree Model

The two-step response process for answering a Likert item captured by the decision tree in Figure 2 can be modeled using an IRTree model. Let *X*_*pi*_ be the random variable for the item score of person *p* on item *i*, with realizations *x*_*pi*_ = 0, …, *m*, …, *h*, where *h* is even and $m=\frac{h}{2}$ . Let *Z*_*pi*_ be a dichotomous variable that takes on the value 0 if the response terminated at the first node, and 1 else (i.e., if the response terminated at the second node). *Z*_*pi*_ thus indicates whether the response that was provided was intended to be informative. For *X*_*pi*_ ≠ *m*, *Z*_*pi*_ = 1 and hence is observed, and for *X*_*pi*_ = *m*, *Z*_*pi*_ is either 0 or 1, and is unobserved. Furthermore, let ***θ***_*p*_ = {*θ*_*p*1_, *θ*_*p*2_} be a vector of latent variables of person *p*, where *θ*_*p*2_ is the latent variable that the scale was intended to measure (i.e., the latent variable that is part of the second node of the IRTree), and *θ*_*p*1_ captures a non-response tendency (i.e., the latent variable operating at the first node of the IRTree).

We can now consider a general expression for the probability of obtaining a particular outcome *x*_*pi*_:

(1) $$P\left(X_{pi}=x_{pi}|\theta_{p}\right)=P\left(X_{pi}=x_{pi}|\theta_{p},Z_{pi}=1\right)P\left(Z_{pi}=1|\theta_{p}\right)+P\left(X_{pi}=x_{pi}|\theta_{p},Z_{pi}=0\right)P\left(Z_{pi}=0|\theta_{p}\right).$$

Furthermore, for *x*_*pi*_ ≠ *m*

(2) $$P\left(X_{pi}=x_{pi}|\theta_{p},Z_{pi}=0,x_{pi}\neq m\right)=0,$$

and hence

(3) $$P\left(X_{pi}=x_{pi}|\theta_{p},x_{pi}\neq m\right)=P\left(X_{pi}=x_{pi}|\theta_{p},Z_{pi}=1\right)P\left(Z_{pi}=1|\theta_{p}\right).$$

Since *P*(*X*_*pi*_ = *m*|*Z*_*pi*_ = 0) = 1, for *x*_*pi*_ = *m* we obtain

(4) $$P\left(X_{pi}=m|\theta_{p}\right)=P\left(Z_{pi}=0|\theta_{p}\right)+P\left(X_{pi}=m|Z_{pi}=1,\theta_{p}\right)P\left(Z_{pi}=1|\theta_{p}\right).$$

Thus, the probability that a middle-category response that has been provided is informative corresponds to

(5) $$P\left(Z_{pi}=1|X_{pi}=m,\theta_{p}\right)=\frac{P\left(X_{pi}=m,Z_{pi}=1|\theta_{p}\right)}{P\left(X_{pi}=m|\theta_{p}\right)},$$

where

(6) $$P\left(X_{pi}=m,Z_{pi}=1|\theta_{p}\right)=P\left(Z_{pi}=1|\theta_{p}\right)P\left(X_{pi}=m|Z_{pi}=1,\theta_{p}\right).$$

Consequently, only the functions *P*(*Z*_*pi*_ = 1| ***θ***_*p*_) = *P*(*Z*_*pi*_ = 1| *θ*_*p*1_) and *P*(*X*_*pi*_ = *x*_*pi*_| ***θ***, *Z*_*pi*_ = 1) = *P*(*X*_*pi*_ = *x*_*pi*_ | *θ*_*p*2_, *Z*_*pi*_ = 1) need to be specified for one to be able to determine the probability of observing a particular response. This can be done using common IRT models.

One can opt to describe the probability of obtaining a certain response in the second node of the IRTree model (i.e., given *Z*_*pi*_ = 1) using a common polytomous IRT model, such as the graded response model (GRM; Samejima, 1969). In the GRM the cumulative probabilities are modeled:

(7) $$P\left(X_{pi}\geq k\;|\;\theta_{p2},Z_{pi}=1,\alpha_{2i},\delta_{i}\right)=\Phi \left(\alpha_{2i}\theta_{p2}-\delta_{ik}\right),\forall k\in \left[1:h\right],$$

where Φ(⋅) denotes the standard normal distribution function, *α*_2*i*_ is the slope of item *i* in the second node which determines the strength of the relationship between the measured latent variable and the item score, and *δ*_*ik*_ is the *k*-th threshold of item *i*, with ***δ***_*i*_ = {*δ*_*i*1_, …, *δ*_*ik*_, …, *δ*_*ih*_}. Hence, in the second node the probability of obtaining the score *x*_*pi*_ is

(8) $$P\left(X_{pi}=x_{pi}|\theta_{p2},\;Z_{pi}=1,\;\alpha_{2i},\;\delta_{i}\right)=\left\{\begin{array}{c} 1-\Phi \left(\alpha_{2i}\theta_{p2}-\delta_{i1}\right),\text{ if }x_{pi}=0;\;\\ \Phi \left(\alpha_{2i}\theta_{p2}-\delta_{ik}\right)-\Phi \left(\alpha_{2i}\theta_{p2}-\delta_{i\left(k+1\right)}\right),\text{ if }x_{pi}=k\in \left[1\;:\;\left(h-1\right)\right];\;\\ \Phi \left(\alpha_{2i}\theta_{p2}-\delta_{ih}\right),\text{ if }x_{pi}=h.\; \end{array}\right.$$

Likewise, we can opt to model the process at the first node using a common dichotomous IRT model, for example, the normal-ogive model (Lord & Novick, 1968). In that case

(9) $$P\left(Z_{pi}=1\;|\;\theta_{p1},\alpha_{1},\beta_{i}\right)=\Phi \left(\alpha_{1}\left(-\theta_{p1}\right)-\beta_{i}\right),$$

where *α*_1_ ≥ 0 is the common item slope relating the probability of providing an informative response to the person’s non-response tendency *θ*_*p*1_, and (−*β*_*i*_) is the intercept of item *i*. Here, −*θ*_*p*1_ rather than *θ*_*p*1_ is used, such that *θ*_*p*1_ refers to a non-response tendency, with higher values on *θ*_*p*1_ meaning that a person is less likely to give an informative response. By allowing items to differ in their intercept, it is acknowledged that some items will be more prone to non-response usage of the middle response category than others. Since *α*_1_ captures the overall item slope, it indicates to what extent non-response usage of the middle response category can be explained by *θ*_*p*1_, with a high value of *α*_1_ suggesting that persons differ markedly in the frequency with which they provide uninformative responses.

## Estimation

We propose to estimate the model by obtaining samples from the joint posterior distribution of the model parameters: For each parameter the mean of the sampled values can be used as its point estimate, and the 95% credible interval can be used to quantify the uncertainty about its value. The joint posterior distribution is proportional to the product of the density of the data and the prior distribution. The density of the data is the following

(10) $$\begin{array}{l} P\left(X=x\;|\;\theta ,\alpha_{1},\alpha_{2},\beta ,\delta \right)=\\ \overset{N}{\underset{p=1}{\prod }}\overset{K}{\underset{i=1}{\prod }}\left(I\left(x_{pi}=m\right)P\left(Z_{pi}=0|\theta_{p1},\alpha_{1},\beta_{i}\right)+P\left(Z_{pi}=1|\theta_{p1},\alpha_{1},\beta_{i}\right)P\left(X_{pi}=x_{pi}|\theta_{p2},\;Z_{pi}=1,\;\alpha_{2i},\;\delta_{i}\right)\right), \end{array}$$

where **X** is an *N* × *K* matrix containing the item scores of the *N* persons on the *K* items, ***θ*** is an *N* × 2 matrix containing the latent variables of all persons, ***α***_2_ is a vector of length *K* containing the slope parameters for the second node of all items, ***β*** is a vector of length *K* containing the intercepts in the first node of all items, ***δ*** is a *K* × (*h* − 1) matrix containing the threshold parameters for the second node of all items, and where I(⋅) denotes the indicator function.

When choosing priors we aimed at including as little information as possible and let the posterior distribution be dominated by the data. The prior information that is included is very limited and is meant to make sure that the posteriors are proper, the parameters take on values that are typically observed for the parameters of IRT models, and that the necessary constraints on the parameter values are satisfied. Where possible we chose semi-conjugate priors to simplify the shape of the conditional posteriors.

For the person parameters, we use a bivariate normal prior distribution, which is common for multidimensional IRT models and results in normal conditional posterior distributions for the person parameters. For identification the mean vector needs to be constrained to 0, and the variances need to be constrained to 1. However, to speed up convergence we sample the mean ***μ*** and the covariance matrix **Σ** freely, but for each sample from the posterior re-scale the parameters such that the means of the latent variables are equal to 0 and the variances are equal to 1. For ***μ*** we use an improper prior (∝ 1), and for **Σ** we use a semi-conjugate inverse-Wishart prior with four degrees of freedom and the identity matrix as a scale parameter. Such a choice of the parameters of the prior makes sure that the posterior distribution is dominated by the data when the sample size is large.

For the item parameters we use independent low-informative priors

(11) $$\begin{array}{l} f\left(\alpha_{1},\alpha_{2},\beta ,\delta \right)\propto N\left(\alpha_{1};0.5,4\right)I\left(\alpha_{1}\geq 0\right)\times \\ \overset{K}{\underset{i=1}{\prod }}N\left(\beta_{i};-2,4\right)N\left(\alpha_{2i};0.5,4\right)I\left(\alpha_{2i}\geq 0\right)I\left(-5\leq \delta_{i1}\leq \ldots \leq \delta_{i\left(h-1\right)}\leq 5\right). \end{array}$$

For the intercept and the slope parameters semi-conjugate priors are used. Here, *α*_1_ and all *α*_2*i*_s are constrained to be positive to ensure clear interpretation of the person parameters *θ*_*p*1_ and *θ*_*p*2_, and the indicator function $I\left(-5<\delta_{i1}<\ldots <\delta_{i\left(h-1\right)}<5\right)$ is used to ensure that the threshold parameters fall within reasonable bounds and are placed in the correct order. The priors are chosen to be independent since we do not have any particular expectations about how the different parameters would be related to each other.

To obtain estimates of all relevant parameters a Gibbs sampler (Geman & Geman, 1984) was implemented in R (R Core Team, 2016). In the Gibbs Sampler, the parameters are consecutively sampled from their conditional posterior distribution given the current values of other parameters. To simplify the conditional posteriors we use data augmentation (Tanner & Wong, 1987): For each middle-category response *Z*_*pi*_ is sampled along with the model parameters, for each combination of an item and a person augmented continuous responses $Y_{\mathrm{pi1}}∼N\left(-\alpha_{1}\theta_{p1}-\beta_{i},1\right)$ and $Y_{\mathrm{pi2}}∼N\left(\alpha_{i2}\theta_{p2},1\right)$ are sampled (Albert & Chib, 1993), respectively, matching the first and the second node of the IRTree. The specification of the steps of the Gibbs Sampler can be found in the online supplemental materials.

## Simulation Study

A simulation study was performed with two goals: To investigate the parameter recovery under the new response-mixture model (RMM), and to explore the impact of modeling (RMM) versus ignoring (GRM) the possible differential use of the middle category by the respondents for the estimates of the person and the item parameters.

### Method

The influence of the following three factors on parameter recovery was investigated: sample size (*N* = 1000, 2000), number of items (*K* = 25, *K* = 50), and the overall proportion of non-informative-responses (NR-rate; 20%, 10%, and 0%). A full factorial 2 × 2 × 3 design was used. The conditions with no non-informative responses were included to investigate the results for the RMM when in fact all respondents always interpret the middle category as a neutral position between “agree” and “disagree,” and served as a check against overfitting. All items consisted of 5 categories.

On the person side, *θ*_*p*1_ and *θ*_*p*2_ of each person were sampled independently from the standard normal distribution. Person parameters were generated for 1000 persons, which were used for all replications and all conditions. For the conditions with *N* = 2000 the same set of person parameters was used twice.

On the item side, parameters were generated for 25 items, which were used once in the condition where *K* = 25 and twice in the condition where *K* = 50. The same item parameters were used in all conditions with the exception of the intercept parameters in the first node (***β***), since these determine the proportion of non-informative responses. These intercept parameters were sampled once per non-response condition from a normal distribution with a standard deviation of 0.2 and the mean chosen such that the overall proportion of non-responses is about 20% (mean of −1.6) or 10% (mean of −2.4). For the conditions with no non-informative responses, the intercepts in the first node were set equal to −1000. The slope parameter in the first node of the IRTree was equal to .61, which corresponds to the average discrimination parameter of a “good test” as considered by Reise and Yu (1990).

For the second node, the item threshold parameters were obtained in the following way. First, a general location parameter *γ*_*i*_ was assigned to each of the 25 items, for which equidistantly spaced values between −1 and 1 were used. Second, for each item the four threshold values were sampled from uniform distributions in the following ranges: [−2,−1], [−1,0], [0,1], [1,2]. Only such sets of four values for which the distance between each pair of thresholds was larger than 0.5 were retained. Third, the location *γ*_*i*_ was added to each of the threshold parameters of item *i*. The item slope parameters in the second node of the IRTree were sampled from a uniform distribution between 0.44 and 0.78, matching the range specified by Reise and Yu (1990) for a good test.

For each condition 100 data sets were generated under the RMM. For each data set the RMM and the traditional GRM were estimated using Gibbs Samplers (see online supplemental materials for details) with 2500 iterations (500 burnin, retaining every second iteration after burnin). The number of iterations was chosen based on a pilot study. For the parameters that are of primary interest (*θ*_2_, ***α***_2_, and ***δ***; i.e., the parameters shared by the GRM and RMM) the mean absolute bias, variance, and mean squared error of the point estimates (i.e., posterior means) under the two models were computed.

### Results

Table 1 displays the results of the simulation study for the recovery of *θ*_*p*2_, the latent trait that the test was intended to measure. Here it can be observed that under the GRM a higher proportion of non-informative responses (i.e., higher NR-rate) results in both an increase of the average absolute bias and of the variance of the estimates, and consequently also an increase in the MSE. With respect to the average absolute bias, the impact of increasing the proportion of non-responses is especially strong for the longer test (*K* = 50), where with 20% non-informative responses the average absolute bias for the GRM is more than twice as large as it is with 0% non-informative responses. As can be expected, shorter tests have higher absolute bias and variance of the estimates than longer tests. Sample size did not notably effect any of the outcome measures.

**Table 1.** Average Absolute Bias, Variance, and Mean Squared Error for *θ*_*p*2_ Under the GRM and the RMM, Based on 100 Replications and Computed Using the Posterior Means of the Parameters.

			GRM			RMM		
*N*	*K*	NR-rate	Bias	Var	MSE	Bias	Var	MSE
1000	25	0%	0.090	0.111	0.125	0.090	0.111	0.125
		10%	0.108	0.122	0.154	0.102	0.122	0.143
		20%	0.142	0.136	0.196	0.121	0.139	0.169
	50	0%	0.057	0.074	0.079	0.057	0.075	0.080
		10%	0.081	0.083	0.103	0.066	0.085	0.093
		20%	0.129	0.089	0.144	0.070	0.092	0.103
2000	25	0%	0.098	0.120	0.136	0.098	0.120	0.136
		10%	0.111	0.127	0.164	0.106	0.128	0.152
		20%	0.137	0.128	0.183	0.113	0.131	0.157
	50	0%	0.060	0.077	0.083	0.060	0.078	0.083
		10%	0.084	0.084	0.110	0.066	0.085	0.094
		20%	0.127	0.090	0.138	0.073	0.093	0.104

For the RMM, it can be observed that when there are no non-informative responses (NR-rate = 0) the results are practically equivalent to those obtained under the GRM. Hence, even though the RMM is more complex than the GRM, there does not appear to be a risk of overfitting. For nonzero proportions of non-informative responses (NR-rate >0 ) differences between the two models appear in terms of the bias of the estimates: With more non-informative responses the difference in absolute bias increases. The difference between the two models become more pronounced for longer tests (*K* = 50), where more information is available to estimate *θ*_*p*1_ (the latent trait in the first node) and hence where under the RMM more information is available to determine whether a particular response was informative or not. For *K* = 50 and 20% non-informative responses, the average absolute bias decreases by 44% to 45% when using the RMM rather than the GRM, and the MSE decreases by about 24% to 28%. As the variance of the estimates is comparable for both models, the reduction in bias observed when the RMM is used instead of the GRM does not seem to be at the cost of any notable increase in variance.

**Figure 3.**
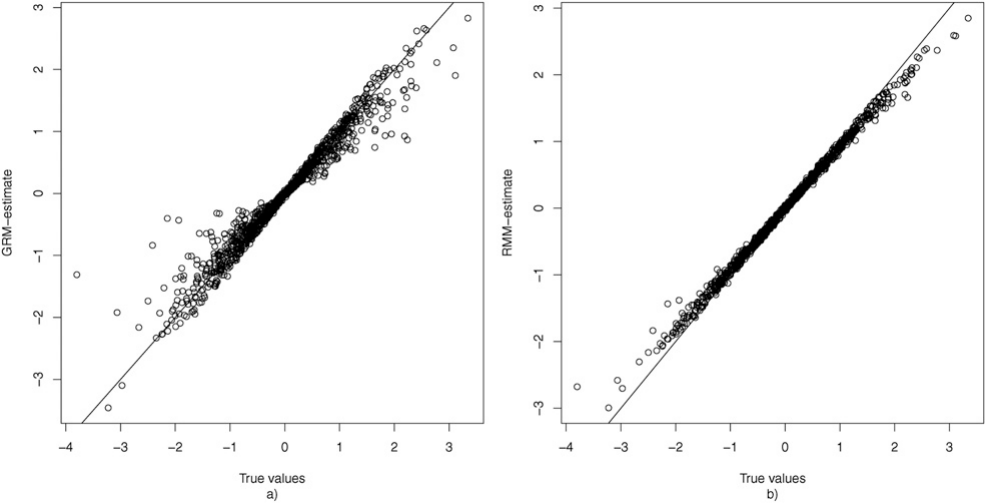
The true values of *θ*_2_ plotted against the average estimates (based on 100 replications) obtained under the GRM (a) and RMM (b) for the condition with *K* = 50, *N* = 1000, and 20% non-informative responses.

To gain insight into the extent to which estimation of *θ*_*p*2_ is confounded when one uses the GRM when some persons use the middle response category as a non-response option, Figure 3(a) displays the true value of all *θ*_*p*2_s plotted against their mean estimate obtained under the GRM over the 100 replications. This figure shows that ignoring the fact that some responses may not have been informative results in a shrinkage of the estimate of *θ*_*p*2_ towards 0. As persons differ in their non-response tendency (*θ*_*p*1_), this shrinkage is stronger for some persons than for others.

Figure 3(b) plots the true value of each *θ*_*p*2_ against their mean estimates of *θ*_*p*2_ as obtained based on the RMM. Here it can be observed that the estimates are located much closer to the identity line, and hence that the average bias is much smaller than under the GRM. Additionally, there are no notable outliers in terms of bias, meaning that unlike under the GRM there were no respondents for whom their *θ*_*p*2_-value was wildly over- or underestimated. It may be noted that also under the RMM there appears to be some shrinkage of the estimates towards 0. However, this is to be expected since 0 is the mean of the hierarchical prior distribution of *θ*_2_, and such shrinkage can be considered desirable as it reduces prediction error (Fox, 2010).

**Figure 4.**
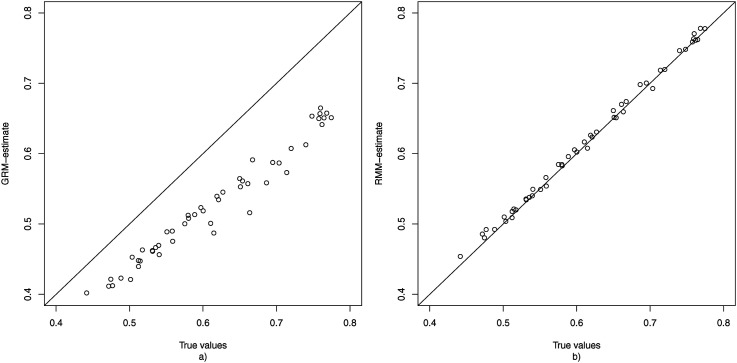
The true values of ***α***_2_ plotted against the average estimates (based on 100 replications) obtained under the GRM (a) and RMM (b) for the condition with *K* = 50, *N* = 1000, and 20% non-informative responses.

To evaluate the recovery of the item parameters, the average estimate of each parameter shared by the GRM and the RMM was plotted against its true value for the condition where *K* = 50, *N* = 1000, and 20% non-informative responses. Figure 4 shows the results for ***α***_2_. Under the GRM the discrimination parameter of each item is notably underestimated, meaning that the results suggest that the items are of lower quality (i.e., less informative of *θ*_*p*2_) than they really are. This can be explained by the fact that 20% of the responses (and hence a large part of all middle-category responses) were not informative and hence constitute noise that dampens the discriminatory power of each item. Under the RMM, this noise is effectively filtered out in the first node, and as a result the estimates of the discrimination parameters in the second node appear to be practically unbiased.

**Figure 5.**
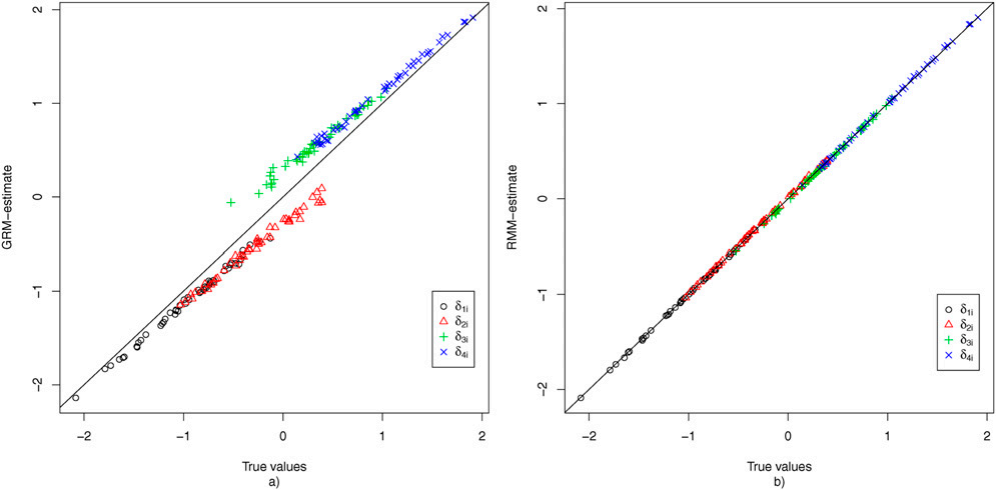
The true values of ***δ*** plotted against the average estimates (based on 100 replications) obtained under the GRM (a) and RMM (b) for the condition with *K* = 50, *N* = 1000, and 20% non-informative responses.

Figure 5 displays the bias for each of the threshold parameters for the condition where *K* = 50, *N* = 1000, and 20% non-informative responses, under both the GRM and the RMM. The results for the GRM show that for all items the first two threshold parameters are underestimated, while the second two threshold parameters are overestimated. This can be explained by considering the fact that due to the middle response category also being used as a non-response option, the proportion of observed middle-category responses is increased, which pushes the first two threshold parameters apart from the last two threshold parameters. Under the RMM, the non-informative usage of the middle response category is filtered out at the first node, and hence all threshold parameters in the second node appear to be recovered without any bias.

## Empirical Example

To investigate the relevance of using the proposed RMM in practice, the procedure was applied to data from a personality questionnaire measuring Machiavellianism (Christie & Geis, 2013). The test consisted of 20 5-category Likert items, which presented the respondents with statements such as “Anyone who completely trusts anyone else is asking for trouble,” to which they had to rate their degree of agreement. The middle response category functioned as the neutral response option, with the label “neutral.” A subset of 2000 respondents was randomly selected based on a larger data set to obtain a sample size that is more representative for typical applications.

**Figure 6.**
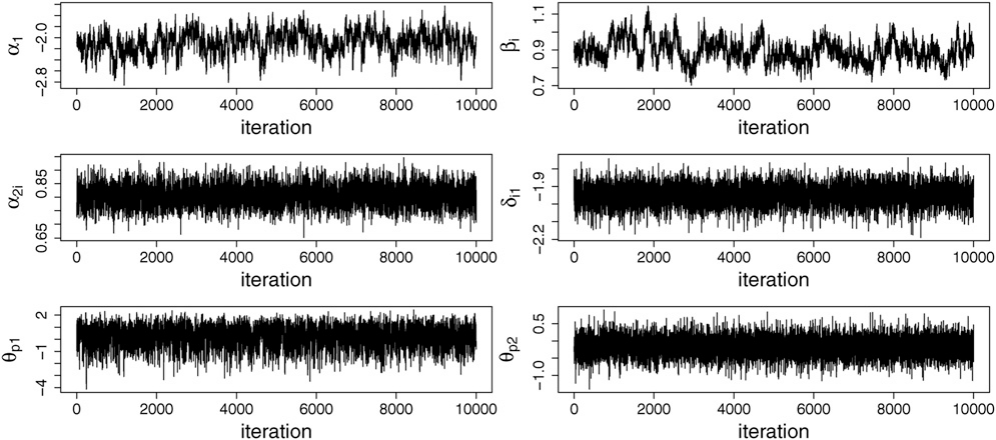
Trace plots for the parameters of the response mixture model (common slope parameters in the first node, *α*_1_, for one of the items: the intercept in the first node, *β*_*i*_, the slope in the second node, *α*_2*i*_, and one of the thresholds in the second node, *δ*_*i*1_, for one of the persons: the person parameter in the first node, *θ*_*p*1_ and the person parameter in the second node, *θ*_*p*2_).

The RMM was fitted to the data together with two alternative models: the GRM (i.e., all middle category responses are considered informative for the measured construct) and the IRTree model (i.e., all middle category responses are considered non-informative for the measured construct; see Figure 1(a)). All three models were fitted to the data using 25,000 iterations in the Gibbs sampler, of which the first 5000 were discarded as burnin. With respect to the number of iterations, we decided to stay on the safe side compared to the simulation study by taking both a longer burn-in and using more iterations for the post-burn-in, because computational time is less of an issue when only one data set needs to be analyzed. The same as in the simulation study, every second iteration after the burnin was retained and used for the computation of the summaries of the posterior distribution. Thinning was performed to decrease the effect of autocorrelation in the Markov chain on the evaluation of posterior variances and credible intervals of the parameters. Convergence was assessed through visual inspection of the trace plots of the model parameters, some of which are displayed in Figure 6 and which did not suggest any major issues with convergence.

To determine which of the three models should be preferred for this dataset, the deviance information criterion (DIC; Spiegelhalter et al., 2002) was considered. The DIC balances model fit and complexity, and can be used for model selection in Bayesian data analysis. For the RMM and the IRTree model, the deviance was computed by integrating out *θ*_1_ using Gauss-Hermite quadrature with 10 nodes. The DIC for the GRM was 103,842, the DIC of the IRTree model was 110,619, and the DIC of the RMM was 103,579, indicated that the RMM should be preferred. Thus, the results suggest that it is not optimal to assume that all middle category responses are informative responses nor is it optimal to assume that all middle category responses are non-informative, but rather that the middle response category has both been used as an informative and as a non-informative response option.

Under the RMM, estimates are obtained for the item parameters in the first node of the tree. Here, the slope parameter is estimated to be 0.89 (with the 95% credible interval ranging from 0.78 to 1.03), which suggests that the items differentiate rather well between persons with high and low *θ*_*p*1_, considering the fact that the items were not designed to measure a non-response tendency. The average of the estimated *β*_*i*_s was −2.33, with −3.20 and −1.78 as the lowest and the highest estimate, respectively. This suggests that none of the items receive a very high proportion of non-informative responses, but that items do differ in the extent to which they evoke non-informative responses.

As for each middle-category response the model provides a posterior probability of that response being non-informative (*P*(*Z*_*pi*_ = 0 | **X**)), the overall proportion of non-informative responses can be estimated by considering the average of *P*(*Z*_*pi*_ = 0 | **X**) over all items and persons. This average is 4.91%, suggesting that about 1 in 20 responses is non-informative. Of all middle-category responses, 30.76% are estimated to be non-informative. Hence, even though there appears to be a non-negligible proportion of non-informative responses, most of the middle-category responses do appear to be informative for this dataset.

On the person side, the correlation between *θ*_1_ and *θ*_2_ was estimated to be −.19 (with the 95% credible interval ranging from −.28 to −.10). This suggests a small negative association between the substantive trait that the test was intended to measure—Machiavellianism—and the non-response tendency. As lower values on *θ*_*p*1_ correspond to being more inclined to giving an informative response, this would indicate that persons who display a strong Machiavellian personality are more inclined to provide informative answers. However, given the size of the correlation this relationship does not appear to be very strong.

## Discussion

This paper presented an IRTree model with a mixture component for observed middle-category responses, which allows users to both correct for and model the possible usage of the middle response category as a non-informative response option. The RMM provides an important addition to the literature, as it has several advantages that can be relevant in practice, where this non-response usage is likely to occur on many questionnaires. First, it purifies the estimation of the person parameters of interest, as a source of confounding is removed. Second, it purifies the estimation of the item parameters, which are likely to become biased if not all responses were intended to be informative. As the simulation study showed, adequately modeling this non-response usage may lead to higher estimated discrimination parameters and hence higher assessed item quality. Third, at the person level information is obtained about each person’s non-response tendency, which can be of substantive interest for researchers. For example, it may be interesting to investigate why Machiavellianism appears to be related to this non-response tendency, as was observed in the empirical example. Fourth, information about each item’s disposition to evoke non-informative responses is obtained. This may both be helpful for the analysis of that particular test (i.e., finding problematic items) and for future test construction (e.g., determining what type of items often evoke non-informative responses). Finally, the percentage of non-informative responses can be evaluated at the person-, item-, and test-level, which may be relevant information for test administrators.

The RMM that was presented in this paper is designed for the analysis of a single unidimensional scale. It would be relevant to extend the current model to a multidimensional framework, such that it can in a single analysis deal with multiple scales measuring different attributes. A benefit of being able to apply the model to multiple scales would be that more information is available for the estimation of the non-response tendency *θ*_*p*1_. As the simulation study suggests that the benefits of using the RMM to correct for persons’ non-response tendencies are especially notable for larger tests, this extension to the multidimensional setting would likely further increase the added value of the RMM in practice, as it may make it feasible to apply the model to a set of short scales.

It should be noted that the current model is explicitly designed to specifically differentiate between informative and non-informative usage of the middle response category. The model is not designed to differentiate between different informed uses of the middle response category, and will as such also not be an adequate tool for analyzing between-person differences in how inclined a person is to use the middle response category to represent their position. Thus, the model does not capture a midpoint responding tendency or the absence of an extreme responding tendency, both of which relate purely to the informed usage of the middle response category. Modeling such between person differences in the informed usage of the middle response category will require the use of psychometric models specifically designed to capture those specific response styles, such as for example many of the models considered in the Introduction. Each of those models will differ in the exact assumptions that they make about what kind of response style is considered to possibly be present, and practitioners should consider carefully which specific response styles they consider plausible or relevant for a given application. The main settings in which the current model can be considered relevant are those settings in which practitioners would consider it plausible that a non-negligible proportion of the respondents have selected the middle response option as a non-response option. With this in mind, the model might be especially relevant for low-stake testing settings, as well as for tests that contain items to which not all respondents will be able to provide a meaningful response (e.g., due to question difficulty or a lack of required background knowledge).

While in its current form the RMM only considers a non-informative-response tendency, one may consider further extending the model to also capture between person differences in the informed usage of the middle response category, as well as other response styles. For example, one could replace the unidimensional GRM in the second node by a more complex model that incorporates extreme response style (e.g., see Tutz & Berger, 2016), to take into account that persons who have decided to give an informative response may still differ in how willing they are to consider extreme versus non-extreme response options. However, as adding further layers to the model increases its complexity, using such extended models may require relatively large sample sizes, and the parameter recovery of such extended models would warrant further study.

## Supplemental Material

Supplemental Material - Modeling Within- and Between-Person Differences in the Use of the Middle Category in Likert Scales Supplemental Material for Modeling Within- and Between-Person Differences in the Use of the Middle Category in Likert Scales by Jesper Tijmstra, and Maria Bolsinova in Applied Psychological Measurement

## Note

1. It may be noted that the mixture component is only needed for dealing with observed middle-category responses, and hence that only for those responses mixture modeling is needed. We will use the term “response-mixture model” to refer to this model, both to keep the terminology manageable and to make the relevant differences with other approaches—where the mixture modeling is at the person level—clear.
