# Supplementary material for: Modeling Within- and Between-Person Differences in the Use of the Middle Category in Likert Scales
Source: Appl Psychol Meas. 2025 Mar 2;49(6):266–81. doi: 10.1177/01466216251322285 (PMC11873858; doi:10.1177/01466216251322285)
Supplement: Supplemental Material - Modeling Within- and Between-Person Differences in the Use of the Middle Category in Likert Scales [file sj-zip-1-apm-10.1177_01466216251322285.zip › Response_mixture_IRT_for_Likert_scales (35).pdf]

Supplementary materials: Steps of the Gibbs Sampler for estimating the response mixture model

Before describing the steps of the Gibbs Sampler we describe the specification of the initial values for the parameters. For all middle-category responses Z_{pi} is sampled from $Bernoulli(.5)$, all person parameters are sampled from $\mathcal{N}(0,1)$, all slope parameters are set to be equal to 0.1, all β_is are sampled from $\mathcal{N}(-1.5, 0.5^2)$, all $\boldsymbol{\delta}_i$s are set to be equal to $\{-1.5, -0.5, 0.5, 1.5\}$, and $\boldsymbol{\Sigma}$ is set to be equal to the identity matrix. After initialization the algorithm repeatedly goes through the following steps:

Step 1. For each combination of person p with item i sample the augmented response Y_{pi1} in the first node of the IRTree:

$$Y_{pi1} \sim \mathcal{N}(-\alpha_1\theta_{p1} - \beta_i, 1)(\mathcal{I}(Y_{pi1} \geq 0))^{Z_{pi}}(\mathcal{I}(Y_{pi1} < 0))^{1-Z_{pi}}. \tag{1}$$

Step 2: For each person p sample the person parameter in the first node of the IRTree:

$$\theta_{p1} \sim \mathcal{N}\left(\frac{-\frac{\sum_i \alpha_1(Y_{pi1}+\beta_i)}{K\alpha_1^2} + \frac{\Sigma_{1,2}\theta_{p2}}{1-\Sigma_{1,2}^2}}{\frac{1}{K\alpha_1^2} + \frac{1}{1-\Sigma_{1,2}^2}}, \frac{1}{\frac{1}{K\alpha_1^2} + \frac{1}{1-\Sigma_{1,2}^2}}\right). \tag{2}$$

Step 3: Sample the slope in the first node of the IRTree:

$$\alpha_1 \sim \mathcal{N}\left(\frac{-\frac{\sum_p\sum_i \theta_{p1}(Y_{pi1}+\beta_i)}{K\sum_p \theta_{p1}^2} + \frac{0.5}{0.25}}{\frac{1}{K\sum_p \theta_{p1}^2} + \frac{1}{0.25}}, \frac{1}{\frac{1}{K\sum_p \theta_{p1}^2} + \frac{1}{0.25}}\right)\mathcal{I}(\alpha_1 > 0). \tag{3}$$

Step 4: For each item i sample the intercept in the first node of the IRTree:

$$\beta_i \sim \mathcal{N}\left(\frac{\frac{\sum_p (Y_{pi1}-\alpha_1\theta_{p1})}{N} + \frac{-2}{4}}{\frac{1}{N} + \frac{1}{4}}, \frac{1}{\frac{1}{N} + \frac{1}{4}}\right). \tag{4}$$

Step 5: For each combination of person p with item i for which $Z_{pi} = 1$, sample the

augmented response Y_{pi2} in the second node of the IRTree:

$$Y_{pi2} \sim \begin{cases} \mathcal{N}(\alpha_{i2}\theta_{p2}, 1)\mathcal{I}(Y_{pi2} < \delta_{i1}), \text{ if } X_{pi} = 0; \\ \mathcal{N}(\alpha_{i2}\theta_{p2}, 1)\mathcal{I}(\delta_{ik} \leq Y_{pi2} < \delta_{i(k+1)}), \text{ if } X_{pi} = k \in [1 : (h-1)]; \\ \mathcal{N}(\alpha_{i2}\theta_{p2}, 1)\mathcal{I}(Y_{pi2} \geq \delta_{ih}), \text{ if } X_{pi} = h. \end{cases} \tag{5}$$

Step 6: For each person p sample the person parameter in the second node of the IRTree:

$$\theta_{p2} \sim \mathcal{N}\left(\frac{\frac{\sum_i Z_{pi}\alpha_{i2}Y_{pi2}}{\sum_i Z_{pi}\alpha_{i2}^2} + \frac{\Sigma_{1,2}\theta_{p1}}{1-\Sigma_{1,2}^2}}{\frac{1}{\sum_i Z_{pi}\alpha_{i2}^2} + \frac{1}{1-\Sigma_{1,2}^2}}, \frac{1}{\frac{1}{\sum_i Z_{pi}\alpha_{i2}^2} + \frac{1}{1-\Sigma_{1,2}^2}}\right). \tag{6}$$

Step 7: For each item i sample the item slope in the second node of the IRTree:

$$\alpha_{i2} \sim \mathcal{N}\left(\frac{\frac{\sum_p Z_{pi}\theta_{p2}Y_{pi2}}{\sum_p Z_{pi}\theta_{p2}^2} + \frac{0.5}{0.25}}{\frac{1}{\sum_p Z_{pi}\theta_{p2}^2} + \frac{1}{0.25}}, \frac{1}{\frac{1}{\sum_p Z_{pi}\theta_{p2}^2} + \frac{1}{0.25}}\right)\mathcal{I}(\alpha_{2i} > 0). \tag{7}$$

Step 8: For each item i consequently sample each of the thresholds δ_{ik}, $k \in [1 : h]$:

$$p(\delta_{ik} \mid \mathbf{X}, \mathbf{Z}, \alpha_{i2}, \boldsymbol{\theta}_1, \boldsymbol{\delta}_i^{(k)}) \propto \mathcal{I}(\delta_{i(k-1)} \leq \delta_{ik} \leq \delta_{i(k+1)}) \prod_i \left(\Pr(X_{pi} = x_{pi} \mid Z_{pi} = 1, \theta_{p2}, \alpha_{i2}, \boldsymbol{\delta}_i)\right)^{Z_{pi}}, \tag{8}$$

where $\boldsymbol{\delta}_i^{(k)}$ are all threshold parameters of item i other than k; $\delta_{i0} \equiv -5$ and $\delta_{i(h+1)} \equiv 5$. Sampling from this conditional posterior is done using a Metropolis-Hastings algorithm with a truncated normal distribution with the mean equal to the current value, a standard deviation of 0.05, a lower bound of $\delta_{i(k-1)}$, and an upper bound of $\delta_{i(k+1)}$. This step is repeated five times to increase the acceptance ratio and improve the mixing of the chain.

Step 9: For each response where $X_{pi} = m$, sample Z_{pi} from

$$Bernoulli\left(\frac{(1-\Phi(-\alpha_1\theta_{p1} - \beta_i))(\Phi(\alpha_{i2}\theta_{p2} - \delta_{i(m-1)}) - \Phi(\alpha_{i2}\theta_{p2} - \delta_{im}))}{\Phi(-\alpha_1\theta_{p1} - \beta_i) + (1-\Phi(-\alpha_1\theta_{p1} - \beta_i))(\Phi(\alpha_{i2}\theta_{p2} - \delta_{i(m-1)}) - \Phi(\alpha_{i2}\theta_{p2} - \delta_{im}))}\right). \tag{9}$$

Step 10: Although the means and the variances of the person parameters are not identified, to improve convergence and to make the estimation more stable the mean vector and the covariance matrix are sampled at each iteration:

$$\boldsymbol{\mu} \sim \mathcal{N}_2\left(\frac{1}{N}\left[\sum_p \theta_{p1}, \sum_p \theta_{p2}\right]^T, \frac{1}{N}\boldsymbol{\Sigma}\right), \tag{10}$$

and

$$\boldsymbol{\Sigma} \sim \mathcal{IW}\left(N+4, \mathbf{I}_2 + \sum_p([\theta_{p1}, \theta_{p2}]^T - \boldsymbol{\mu})([\theta_{p1}, \theta_{p2}] - \boldsymbol{\mu}^T)\right). \tag{11}$$

After $\boldsymbol{\mu}$ and $\boldsymbol{\Sigma}$ are sampled, all parameters are re-scaled according to the identification constraints of $\mu_1 = \mu_2 = 0$ and $\Sigma_{1,1} = \Sigma_{2,2} = 1$:

$$\theta_{pg} \rightarrow \frac{\theta_{pg} - \mu_g}{\sqrt{\Sigma_{g,g}}}, g = \{1, 2\}, p \in [1 : N]; \tag{12}$$

$$\beta_i \rightarrow \beta_i + \mu_1\alpha_1, i \in [1 : K]; \tag{13}$$

$$\alpha_1 \rightarrow \alpha_1\sqrt{\Sigma_{1,1}}; \tag{14}$$

$$\delta_{ik} \rightarrow \delta_{ik} - \mu_2\alpha_{i2}, i \in [1 : K], k \in [1 : h]; \tag{15}$$

$$\alpha_{i2} \rightarrow \alpha_{i2}\sqrt{\Sigma_{2,2}}, i \in [1 : K]; \tag{16}$$

$$\Sigma_{1,2} = \frac{\Sigma_{1,2}}{\sqrt{\Sigma_{1,1}\Sigma_{2,2}}}; \tag{17}$$

$$\Sigma_{1,1} \rightarrow 1; \tag{18}$$

$$\Sigma_{2,2} \rightarrow 1. \tag{19}$$

The same Gibbs Sampler can be used if instead of the RMM one wants to estimate the GRM, but initial values for Z_{pi} should be all equal to 1, Steps 1-4 and 9 should be skipped, and Step 10 should be substituted with the following:

$$\mu_2 \sim \mathcal{N}\left(\frac{1}{N}\sum_p \theta_{p2}, \frac{1}{N}\right), \tag{20}$$

and

$$\Sigma_{2,2} \sim \mathcal{IG}\left(\frac{N}{2}, \frac{\sum_p (\theta_{p2} - \mu_2)^2}{2}\right). \tag{21}$$

After μ_2 and $\Sigma_{2,2}$ are sampled, all parameters are re-scaled according to the identification constraints of $\mu_2 = 0$ and $\Sigma_{2,2} = 1$:

$$\theta_{p2} \rightarrow \frac{\theta_{p2} - \mu_2}{\sqrt{\Sigma_{2,2}}}, p \in [1:N]; \tag{22}$$

$$\delta_{ik} \rightarrow \delta_{ik} - \mu_2 \alpha_{i2}, i \in [1:K], k \in [1:h]; \tag{23}$$

$$\alpha_{i2} \rightarrow \alpha_{i2} \sqrt{\Sigma_{2,2}}, i \in [1:K]. \tag{24}$$
